# Development and evaluation of different normalization strategies for gene expression studies in *Candida albicans *biofilms by real-time PCR

**DOI:** 10.1186/1471-2199-7-25

**Published:** 2006-08-04

**Authors:** Heleen Nailis, Tom Coenye, Filip Van Nieuwerburgh, Dieter Deforce, Hans J Nelis

**Affiliations:** 1 Laboratory for Pharmaceutical Microbiology, Ghent University, Harelbekestraat 72, B-9000, Ghent, Belgium; 2 Laboratory for Pharmaceutical Biotechnology, Ghent University, Harelbekestraat 72, B-9000, Ghent, Belgium

## Abstract

**Background:**

*Candida albicans *biofilms are commonly found on indwelling medical devices. However, the molecular basis of biofilm formation and development is not completely understood. Expression analysis of genes potentially involved in these processes, such as the *ALS *(Agglutinine Like Sequence) gene family can be performed using quantitative PCR (qPCR). In the present study, we investigated the expression stability of eight housekeeping genes potentially useful as reference genes to study gene expression in *Candida albicans *(*C. albicans*) biofilms, using the geNorm Visual Basic Application (VBA) for Microsoft Excel. To validate our normalization strategies we determined differences in *ALS1 *and *ALS3 *expression levels between *C. albicans *biofilm cells and their planktonic counterparts.

**Results:**

The eight genes tested in this study are ranked according to their expression stability (from most stable to least stable) as follows: *ACT1 *(β-actin)/*PMA1 *(adenosine triphosphatase), *RIP *(ubiquinol cytochrome-c reductase complex component), *RPP2B *(cytosolic ribosomal acidic protein P2B), *LSC2 *(succinyl-CoA synthetase β-subunit fragment), *IMH3 *(inosine-5'-monophosphate dehydrogenase fragment), *CPA1 *(carbamoyl-phosphate synthethase small subunit) and* GAPDH *(glyceraldehyde-3-phosphate dehydrogenase).

Our data indicate that five genes are necessary for accurate and reliable normalization of gene expression data in *C. albicans *biofilms. Using different normalization strategies, we found a significant upregulation of the *ALS1 *gene and downregulation of the *ALS3 *gene in *C. albicans *biofilms grown on silicone disks in a continous flow system, the CDC reactor (Centre for Disease Control), for 24 hours.

**Conclusion:**

In conclusion, we recommend the use of the geometric mean of the relative expression values from the five housekeeping genes (*ACT1*, *PMA1*, *RIP*, *RPP2B *and *LSC2) *for normalization, when analysing differences in gene expression levels between *C. albicans *biofilm cells and planktonic cells. Validation of the normalization strategies described above showed that the *ALS1 *gene is overexpressed and the *ALS3 *gene is underexpressed in *C. albicans *biofilms grown on silicone in the CDC reactor for 24 hours.

## Background

*Candida albicans *is an important human fungal pathogen that is associated with biofilm formation on indwelling medical devices like urinary catheters, dental prostheses and silicone voice prostheses [1-3]. Cells released from these biofilms can migrate to the bloodstream and can cause systemic infections [4,5]. Fungal biofilms are highly resistant against most commonly used antimycotics [6]. Since no therapy is able to completely eradicate *C. albicans *biofilms, colonization of these medical devices often results in their functional loss and in most cases necessitates removal and/or replacement of the device [7]. *C. albicans *biofilm formation occurs in different stages of development. Initially, the cells adhere to a surface and form microcolonies. Subsequently, cells in these microcolonies form hyphae and produce an extracellular matrix, which results in a threedimensional structure [8]. The molecular basis of *C. albicans *biofilm formation and development is not completely understood. It is, however, well-established that interaction of *C. albicans *with host cells or inert surfaces leads to changes in gene expression. Different studies have already described changes in gene expression levels during biofilm development [9-11]. For example, Garcia-Sanchez et al. showed that approximately 5% of all *C. albicans *genes are differentially expressed in growing biofilms compared to stationary phase cultured planktonic cells. One of these genes, *ALS1*, was clearly upregulated in *C. albicans *biofilm cells [9]. Using various clinical isolates, O'Connor et al. recently demonstrated that when compared to planktonic cells, the *ALS1 *gene is overexpressed in biofilms formed on silicone elastomer using the absolute quantitative qPCR method [12]. RT (Reverse Transcriptase)-PCR studies of *ALS1 *in *C. albicans *biofilms formed on reconstituted human epithelial cells detected *ALS1 *gene expression over time during the destruction of the epithelium [13]. The *ALS1 *gene belongs to the *ALS *gene family, which encodes cell surface glycoproteins [14]. The *ALS3 *gene, another gene belonging to the ALS gene family has been shown to be upregulated in *C. albicans *hyphae, which suggests that *ALS3 *might also play a role in biofilm development by this organism [15].

Monitoring gene expression by measuring mRNA levels in biofilm and planktonic cells may identify candidate genes involved in biofilm formation. mRNA can be analyzed via different assays such as Northern blot and RT-PCR. However, relative quantitative PCR (qPCR) assays can detect more subtle changes in gene expression, as qPCR data can be normalised against a reference transcript to correct for differences in amount of starting material, RNA integrity, sample to sample variation and RT efficiencies [16,17]. Ideally, reference genes used in relative qPCR studies are housekeeping genes or control genes that are equally expressed in different conditions. Several housekeeping genes have already been used in *C. albicans *gene expression studies, including *ACT1 *(encoding beta-actin), *PMA1 *(plasma membrane ATPase pump) and *TEF1 *(transcript elongation factor) [18-20]. However, numerous studies have shown that the expression of many housekeeping genes is differentially regulated depending on the experimental conditions [21,22]. Recently, Vandesompele et al. showed that the use of multiple control genes results in a much more accurate and reliable normalization of gene expression data [23]. These authors developed a VBA applet called geNorm that allows the determination of the most stably expressed genes from a series of housekeeping genes and of the number of genes required for accurate normalization.

In the present study, we investigated the expression of eight housekeeping genes in *C. albicans *biofilm and planktonic cells by qPCR analysis and subsequent geNorm analysis. To evaluate different normalization strategies we compared *ALS1 *and *ALS3 *gene expression levels in *C. albicans *biofilm and planktonic cells.

## Results

### RNA and cDNA quantity

Different biological replicate samples (Bio A to Bio G and Plankt A to Plankt F for biofilm and planktonic samples, respectively) were obtained after 24 hours of growth, as described in Materials and Methods. Total RNA was extracted from biofilms and planktonic cells (grown in three independent experiments) and cDNA synthesis was performed. The RNA and cDNA concentrations of all the samples are listed in Table 1. The average RNA and cDNA concentrations of the planktonic cells were (mean ± SD) 8.31 ± 1.5 μg/μl and 25 ± 2.61 μg/ml, respectively. The average RNA and cDNA concentrations of the biofilm cells were 4.36 ± 2.91 μg/μl and 18.83 ± 9.45 μg/ml, respectively.

### Standard curves and real time PCR efficiency

Standard curves were generated for all the sample/gene combinations by using the Cycle Threshold (C_t_) value versus the logarithm of each dilution factor. The linear correlation coefficient ranged from 0.9764 to 1.000. Initial amplification efficiencies (E) (obtained using 300 nM primers and 300 nM or 200 nM probe) ranged from 80% to 100%, except for the *CPA1*, *IMH3 *and *LSC2 *genes (E < 70%). Various primer and probe concentrations were retested for those genes, until efficiencies of >80% were obtained (data not shown). Agarose gel-electrophoresis of the amplified products showed a single band of the expected size for each qPCR assay. No primer dimers or non-specific amplification products could be observed. The inter-plate variation was < 0.5 C_t _for every gene tested.

### Expression stability of housekeeping genes

**Figure 1:**
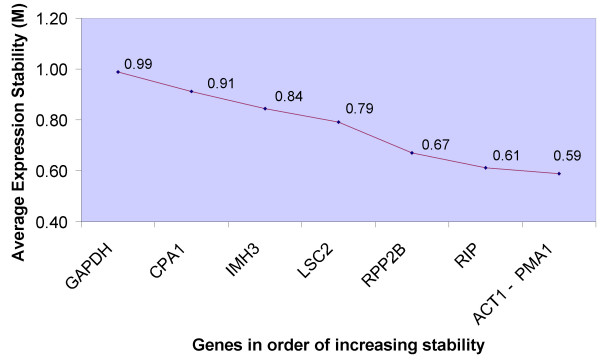
Stability ranking of the housekeeping genes in all samples (biofilms and planktonic cells). Genes are ranked from left to right in order of increasing expression stability (decreasing M value).

**Figure 2:**
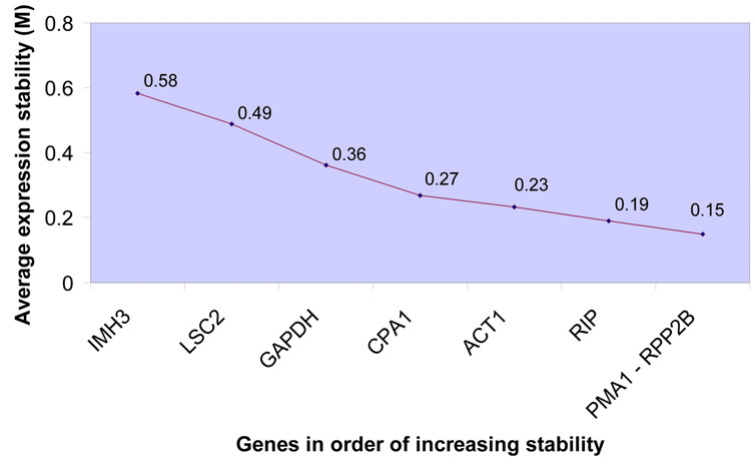
Stability ranking of the housekeeping genes in planktonic cells. Genes are ranked from left to right in order of increasing expression stability (decreasing M value).

**Figure 3:**
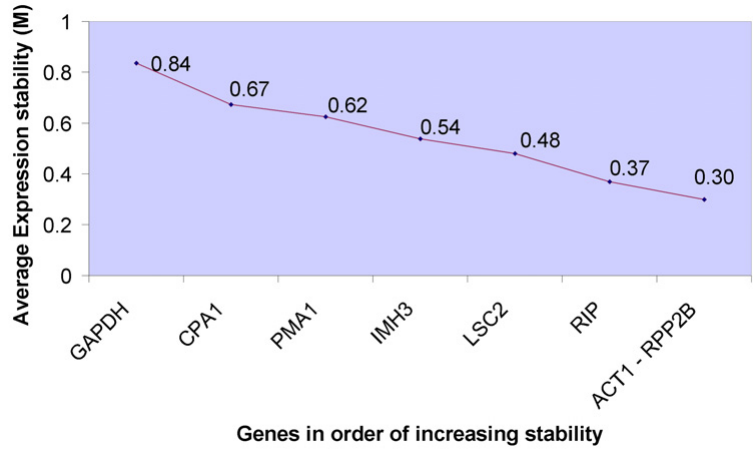
Stability ranking of the housekeeping genes in biofilms. Genes are ranked from left to right in order of increasing expression stability (decreasing M value).

**Figure 4:**
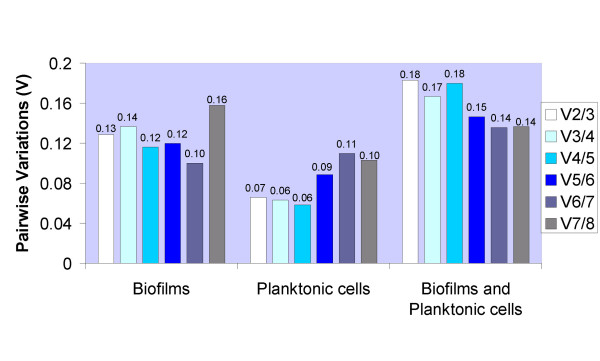
Pairwise variations (V_n_/_n+1_) for all the samples (biofilms and planktonic cells) and biofilms and planktonic cells separately. Pairwise variation between every combination of sequential normalization factors were calculated to determine the minimum number of housekeeping genes required for accurate normalization in the different samples. The cut-off value, below which the inclusion of an additional housekeeping gene does not result in a significant improvement of normalization, was set at 0.15.

The M value of the eight housekeeping (HK) genes for all the samples (biofilm and planktonic cells) and biofilm and planktonic cells separately are listed in Table 2. This M value is defined as the average pairwise variation of one particular gene compared to all the other control genes. The gene with the lowest M value is considered as the most stable gene and the gene with the highest M value is excluded. A new M value is calculated and this procedure is repeated until only two genes are left. These two genes have the lowest M value and are therefore most stably expressed in all the samples. In Fig. 1, 2 and 3, the eight control genes are ranked according to their increasing expression stability (decreasing M value), for all samples (Fig. 1), planktonic cells (Fig. 2) and biofilm cells (Fig. 3). For all samples combined, the genes are ranked from most stable to least stable as follows: *ACT1 *and *PMA1*, *RIP*, *RPP2B*, *LSC2*, *IMH3*, *CPA1 *and *GAPDH *(Fig. 1). No significant improvement in normalization could be observed by using more than five control genes (Fig. 4). When considering biofilms and planktonic cells separately, no significant improvement between the use of two or more than two housekeeping genes was obtained (Fig. 4). The two most stably expressed control genes in *C. albicans *biofilm and planktonic cells separately were the *ACT1 *and *RPP2B *genes, and the *RPP2B *and *PMA1 *genes, respectively.

### ALS1 and ALS3 expression in C. albicans biofilms

cDNA was amplified using a real time PCR MGB (Minor Grooving Binding) Taqman probe assay. The absolute C_t _values from all the qPCR assays were used to calculate the expression ratios of the *ALS1 *and *ALS3 *genes in *C. albicans *biofilm cells compared to their planktonic counterparts. Table 3 shows the mean expression ratios of the *ALS1 *and *ALS3 *genes in *C. albicans *biofilm cells calculated with the four different normalization strategies (i.e., using the five or three most stably expressed HK genes [5HK or 3HK], ACT1 as a control gene and cDNA input standardization, respectively). These ratios are based on the expression of those two genes in all the biofilm samples compared to all the planktonic samples. Statistical analysis shows a significant upregulation of the *ALS1 *gene in *C. albicans *biofilm cells (p < 0.05). When using different normalization methods, we found that the *ALS1 *gene expression was three-fold (5HK genes and cDNA input), four-fold (3HK genes) or five-fold (ACT1) induced in *C. albicans *biofilms compared to planktonic cells. In contrast, there appeared to be a significant downregulation of the *ALS3 *gene in *C. albicans *biofilms (p < 0.05). We found a fourteen-fold (5HK genes), twelve-fold (3HK genes), seven-fold (ACT1) or ten-fold (cDNA input) downregulation of the *ALS3 *gene.

We also compared the *ALS1 *and *ALS3 *gene expression ratios obtained with the four different normalization strategies. No statistically significant difference in *ALS1 *and *ALS3 *gene expression ratios was observed when using the geometric mean of the relative expression levels of three or five housekeeping genes nor with standardization of the cDNA input. However, normalization using *ACT1 *as a single reference transcript resulted in higher expression ratios, both for *ALS1 *and *ALS3 *compared to the other strategies (p < 0.01).

## Discussion

This study describes a comparison between different normalization strategies used in relative RT-qPCR for the quantification of gene expression levels in *C. albicans *biofilm cells, grown on silicone in the CDC reactor, with reference to their planktonic counterparts. We also investigated *ALS1 *and *ALS3 *gene expression levels in *C. albicans *biofilms.

The expression levels of the *ALS1 *gene in *C. albicans *biofilm cells have previously been studied using different techniques, including absolute RT-qPCR, RT-PCR and micro-array analysis [9,12,13]. The choice of an appropriate reference gene for normalization in relative RT-qPCR is critical for accurate and reliable analysis of gene expression data. However, the selection of control genes used for normalization of qPCR data in *C. albicans *is often arbitrary and/or based on observations from previous studies. Commonly used housekeeping genes are the *ACT1 *and *PMA1 *genes [18,20]. However, no single reference gene is expressed constantly in every experimental setup. Transcription levels of control genes used in relative RT-qPCR may vary significantly between different cell types and different developmental stages [22].

Therefore, we empirically validated the expression stability of multiple housekeeping genes in *C. albicans *biofilms and planktonic cells in order to use them as reference transcripts for determining gene expression data. We found that five control genes are required for accurate normalization of gene expression in *C. albicans *biofilms (*ACT1*, *PMA1*, *RIP*, *RPP2B *and *LSC2*). These results suggest that the use of two, three or four control genes significantly alters the normalization factor and thus decreases the reliability and accuracy of gene expression data in *C. albicans *biofilms and planktonic cells. When considering biofilm and planktonic cells separately, we found that the *ACT1 *and *RPP2B *genes (biofilms) and the *PMA1 *and *RPP2B *genes (planktonic cells) were the two most stably expressed genes. There was no significant improvement in the normalization factor when using more than two genes for normalization in biofilm cells and planktonic cells separately. Furthermore, the pairwise variation is much higher in biofilm cells than in planktonic cells, suggesting that the expression levels of the control genes tested in the present study are less stable in biofilm cells than in their planktonic counterparts.

RNA quantity is also a very important factor for accurate and reliable analysis of gene expression data. Therefore, we evaluated the amount of total RNA recovered from biofilm and planktonic cells prior to cDNA synthesis. We found that more total RNA was extracted from planktonic cells than from biofilm cells. We also observed a larger sample-to-sample variation in amount of total RNA for biofilm cells than for planktonic cells. These results suggest that the RNA extraction from biofilm cells is less efficient. To minimize variation of the input RNA in the RT reaction, we decided to standardize the amount of input RNA. This normalization step is essential in the protocol we used (Invitrogen) since the amount of total RNA in the RT reaction is restricted to 5 μg per reaction.

To compare different normalization strategies for gene expression analysis in *C. albicans *biofilms using relative RT-qPCR, we investigated the *ALS1 *and *ALS3 *gene expression levels in *C. albicans *biofilm and planktonic cells. Compared to the other normalization strategies, normalization with *ACT1 *as a control gene gives significantly different results (Table 3). These results clearly indicate the need for a more reliable normalization method. Therefore, we suggest to use five control genes for normalization of gene expression data in *C. albicans *biofilms. For all normalization strategies, we observed a broad range in *ALS1 *and *ALS3 *expression ratios when comparing all "biofilm vs. planktonic" sample combinations (Table 3). Therefore, it is critical to analyze several independent samples to detect small fold changes.

We have chosen to analyze the expression of *ALS1 *and *ALS3 *genes because they encode large glycoproteins implicated in adhesion [15,24,25]. Therefore, these genes could be involved in adhesion to silicone and may contribute to biofilm formation and development on medical devices.

*C. albicans *planktonic cells were harvested during stationary phase. Similarly, biofilm cells were harvested when the biofilm had reached it maximal biomass, i.e. a condition similar to stationary phase (data not shown). Our findings demonstrate that in our model system, the *ALS1 *gene is overexpressed in biofilms compared to their planktonic counterparts. Furthermore, we observed an underexpression of the *ALS3 *gene. Microscopic evaluation revealed that under the conditions used, *C. albicans *forms biofilms with a marked three-dimensional structure consisting of both yeast cells and hyphae. In addition, the planktonic culture contained a lot of hyphae as well. As it was previously shown that *ALS3 *gene expression was upregulated in hyphae [15], this could explain why we could not observe an overexpression of the *ALS3 *gene in biofilm cells. However, differences in expression between the *ALS1 *and *ALS3 *genes may indicate different functions of Als1p and Als3p in *C. albicans *biofilm development.

## Conclusion

This is the first study which validates different normalization methods in order to study changes in fold expression of genes in *C. albicans *biofilms. We suggest to use the geometric mean of five housekeeping genes for the investigation of gene expression in *C. albicans *biofilms compared to planktonic cells. This assay could be useful in the determination of differentially expressed genes in *C. albicans *biofilms compared to their planktonic counterparts. Moreover, this normalization method could be applied to monitor the kinetics of *ALS1 *and *ALS3 *gene expression during biofilm development. Further information about the expression profiles in *C. albicans *biofilms could help to better understand the molecular basis of adhesion to and biofilm formation on silicone and other biomaterials.

## Methods

### Strains and culture conditions

*C. albicans *strain SC5314, which was kindly provided by Dr. A. Brown (Aberdeen University, UK), was used throughout this study. Cells were stored frozen at -80°C using the Microbank system (Prolab Diagnostics). For every experiment a fresh culture was prepared by taking two beads from a Microbank vial and transferring them into 10 ml Sabouraud Dextrose Broth (SDB; BD). This culture was incubated for 24 hours at 37°C. For culturing planktonic cells and biofilms, 50 μl of this suspension was added to 10 ml SDA and incubated for 16 hours at 37°C in a water bath with shaking.

### Biofilm growth

*C. albicans *biofilms were grown on silicone disks (Q7-4735; Dow Corning) in the CDC biofilm reactor (Biosurface Technologies) [26] according to the protocol described by Honraet et al. [27], with some modifications. Cells were harvested by centrifugation, washed three times with and resuspended in 0.9% NaCl w/v. The resulting suspension (containing approximately 10^8 ^CFU/ml) was added to 500 ml Yeast Nitrogen Base (YNB; BD) supplemented with 50 mM glucose. This inoculated medium was transferred to the CDC reactor and incubated at 37°C on a magnetic stir plate (RET digi-visc, IKA Labortechnic) set at 80 rpm for 24 hours, to allow cells to adhere to the silicone disks. After 24 hours, 0.2X YNB supplemented with 10 mM glucose was pumped through the reactor for 24 hours at a flow rate of 400 ml/hour. After 24 hours of biofilm growth, the disks were taken out of the reactor and each disk was transferred to 0.9% NaCl w/v. Prior to RNA extraction, disks were subjected three times to 30 s of sonication (Branson 3510, 42 kHz, 100 W, Branson Ultrasonics Corp.) and 30 s of vortex mixing. This treatment is necessary to remove the biofilm cells from the silicone [28].

### Planktonic growth

After 16 hours of growth, the suspension was centrifuged and the pellets were washed three times with and resuspended in 0.9% NaCl w/v. Subsequently, 40 μl of this suspension was added to 10 ml of YNB broth supplemented with 50 mM glucose and incubated for 24 hours at 37°C in a water bath with shaking. After 24 hours the cells were centrifuged and resuspended in 10 ml diluted YNB (0.2X YNB supplemented with 10 mM glucose). These tubes were incubated for 24 hours at 37°C in a water bath with shaking. After 24 hours incubation, the cells were harvested by centrifugation and resuspended in 0.9% NaCl w/v. Prior to RNA extraction, this 1 ml suspension was subjected to vortex mixing and sonication, as described above.

### RNA extraction and quantification

The cell suspensions were centrifuged and the supernatant was discarded. Total RNA was extracted with the Ambion Pure Yeast kit (Ambion) according to the manufacturer's instructions. Isolated RNA was DNase treated with the DNase treatment kit (Ambion) to ensure the absence of genomic DNA contamination. Further purification of total RNA was carried out with the Microcon columns according to the manufacturer's instructions (Millipore). Total RNA was quantified prior to cDNA synthesis using the RNA Quant-it kit (Invitrogen) according to the manufacturer's instructions.

### cDNA preparation and quantification

First strand cDNA synthesis was performed using the Superscript II kit (Invitrogen). Five μg of total RNA and 1 μl oligodT12-18 primer (0.5 μg/μl) were added to each tube to obtain a total volume of 12.5 μl. Priming was carried out at 70°C for 20 min. Subsequently, 2 μl 10× RT buffer (200 mM Tris-HCl pH 8.4 and 500 mM KCl), 2 μl 25 mM MgCl_2_, 1 μl 10 mM dNTP, 2 μl 0.1 M DTT, 1 μl Superscript II RT (50 units/μl) and 0.5 μl RNaseOut (Recombinant Ribonuclease Inhibitor 40 units/μl) were added to each reaction tube. The RT reaction was carried out at 27°C for 10 min, followed by 50 min at 42°C and finally 5 min at 90°C (Amplitron Thermolyne). Subsequently, 1 μl of *Escherichia coli *RNaseH (2 units/μl) was added to each reaction tube and the tubes were incubated for 20 min at 37°C. cDNA was quantified using the OligoGreen Single Stranded Quantification kit (Invitrogen) according to the manufacturer's instructions.

### Development of primers and MGB Taqman probes

Full-length gene sequences were obtained from the *C. albicans *database [29]. Primers and MGB Taqman probes were designed using the Primer Express software (Applied Biosystems) according to the manufacturer's instructions. The primer and MGB Taqman probe sequences of all the genes used in this study are listed in Table 4. These sequences were compared to the *C. albicans *database using BLAST [30] in order to determine their specificity. Primers and MGB Taqman probes which could have resulted in non-specific signals were excluded. Eight housekeeping genes were selected based on different criteria. First, genes which are commonly used as a control for real time PCR experiments in *C. albicans *were included. Secondly, genes which appeared to be differentially expressed in biofilm and planktonic cells in the micro-array study of Garcia-Sanchez [9] were excluded. Furthermore, to avoid co-regulation of gene expression, the housekeeping genes tested were selected to belong to different functional categories. The control genes tested in this study were *ACT1*, *PMA1*, *RPP2B*, *TDH1 *(*GAPDH*), *LSC2*, *CPA1*, *IMH3 *and *RIP*.

### Real time PCR

Real time PCR was performed in 96 well plates with the ABI 7000 apparatus (Applied Biosystems) using the MGB Taqman probe assay. The concentrations of the primer and MGB Taqman probes used in this study are listed in Table 4. Unless otherwise specified, 5 μl cDNA was added to each reaction. Alternatively, the amount of cDNA was standardized and 40 ng cDNA was added to each reaction. Each reaction contained 12.5 μl Taqman Universal PCR Mastermix in a total volume of 25 μl. The real time PCR reactions were performed at 95°C for 10 min, followed by 40 cycles of 15 s at 95°C and 1 min at 60°C. For each sample a four-point standard curve was made with serial two-fold dilutions (1, 1/2, 1/4 and 1/8). Primer efficiency was determined for every sample/gene combination of all the gene expression assays using the formula *E *= 10^-1/slope^, with "slope" being the slope of the four-point standard curve. Control samples were included on multiple plates to ensure that different plates could be compared. In addition, the samples were subjected to electrophoresis on 2.5 % agarose gels (Invitrogen) in 1× TBE buffer (1 M Trizma Base, 0.9 M boric acid and 10 mM EDTA, pH 8.4; Sigma) for 90 minutes at 100 V. Subsequently, gels were stained with ethidium bromide to confirm the presence of the expected PCR products and the absence of unwanted non-specific products.

### Data analysis using geNorm

Gene stability analysis of different housekeeping genes was performed using the geNorm VBA applet [23]. For every control gene, the expression stability (M) was calculated as the standard deviation of the logarithmically transformed expression ratios. This M value is the average pairwise variation of one particular gene compared to all the other control genes. The gene with the lowest M value is considered as the most stable gene and the gene with the highest M value is excluded. A new M value is calculated and this procedure is repeated until only two genes are left. These two genes have the lowest M value and are therefore most stably expressed in all the samples.

Evaluation of the number of control genes required for accurate and reliable normalization was performed by calculation of the pairwise variation (V) between ranked normalization factors (NF). NFs, based on the expression values of the most stable housekeeping genes (lowest M value), were calculated using the geometric mean. For each combination of two sequential normalization factors (NF_n _and NF_n+1_) the pairwise variation (V_n/n+1_) was calculated (n being the number of housekeeping genes tested). A large variation between two sequential NF's means that the added gene has a significant effect on the calculated normalization factor and should therefore be included in the calculations used for normalizing the data. The cut-off value, below which the inclusion of an additional control gene was considered not to result in a significant improvement of the normalization, was set at 0.15.

### Expression of ALS1 and ALS3 in C. albicans biofilm cells

*ALS1 *and *ALS3 *gene expression in *C. albicans *biofilms was evaluated using four different normalization strategies. Gene expression data were normalized using (i) the geometric mean of the five most stably expressed housekeeping genes (5HK genes), (ii) the geometric mean of the three most stably expressed housekeeping genes (3HK genes), (iii) *ACT1 *gene as a single reference transcript (ACT1), and (iv) standardization of the cDNA input (cDNA input). The normalized expression ratios of the *ALS1 *and *ALS3 *genes using 5HK genes or 3HK genes were calculated according to the procedure described by Vandesompele et al. [23]. Over- or underexpression of these two genes relative to the *ACT1 *gene was calculated as described by Pfaffl [31]. *ALS1 *and *ALS3 *gene expression data obtained by normalization of the input cDNA were calculated using the delta delta C_t _method [32].

### Statistical analysis

Statistical analysis was performed using the SPSS 11.0 software (SPSS). One-way ANOVA was used to compare the four different normalization strategies.

A one-sample two-tailed t-test was used to determine whether differences in *ALS1 *and *ALS3 *gene expression between *C. albicans *biofilms and planktonic cells were statistically significant.

## Authors' contributions

HN participated in the design of the study, performed all the experimental procedures, carried out the data analysis and is the primary author of this manuscript. TC conceived the study, carried out the data analysis and helped to draft the manuscript. DD and FVN participated in the study design. HJN participated in the design and coordination of the study. All authors read and approved the final manuscript.

## Figures and Tables

**Table 1:** RNA and cDNA concentrations of all biofilm and planktonic samples.

**Planktonic samples**	**RNA (μg/μl)**	**cDNA (μg/ml)**	**Biofilm samples**	**RNA (μg/μl)**	**cDNA (μg/ml)**
Plankt A	7.68	22.18	BioA	8.22	10.63
Plankt B	8.32	23.13	BioB	5.80	8.15
Plankt C	11.00	28.25	BioC	1.47	20.78
Plankt D	8.06	27.00	BioD	1.15	18.09
Plankt E	8.23	27.43	BioE	6.57	20.03
Plankt F	7.23	23.27	BioF	2.96	34.56
			BioG	4.30	28.59

**Table 2:** Internal control gene stability value M for all housekeeping genes. A low M value corresponds to a high expression stability of the particular control gene.

**Samples**	**M value for all the housekeeping genes tested**							
	GAPDH	CPA1	IMH3	LSC2	RPP2B	RIP	ACT	PMA1
Biofilms and Planktonic cells	0.99	0.91	0.84	0.79	0.67	0.61	0.59	0.59
Biofilms	0.84	0.67	0.54	0.48	0.3	0.37	0.3	0.62
Planktonic cells	0.36	0.27	0.58	0.49	0.15	0.19	0.23	0.15

**Table 3:** The mean expression ratios of the *ALS1 *and *ALS3 *genes in all the *C. albicans *biofilm samples compared to all the planktonic samples calculated with four different normalization strategies.

**Genes**	**Fold expression calculated with four different normalization strategies**			
***ALS1***	**5HK**	**3HK**	**ACT1**	**cDNA**
Mean	2.98	3.67	5.12	3.35
SD	1.64	2.11	2.33	1.53
Range	0.67–7.54	0.68–8.41	1.85–10.58	1.08–6.57
***ALS3***	**5HK**	**3HK**	**ACT1**	**cDNA**
Mean	0.069	0.083	0.15	0.10
SD	0.02	0.022	0.069	0.033
Range	0.042–0.13	0.044–0.14	0.072–0.35	0.046–0.16

**Table 4:** Primer and MGB Taqman probe sequences and concentrations used in real time PCR.

**Gene symbol**	**Primer/probe**	**Gene Sequence (5'-3')**	**Concentration (nM)**
*ACT1*	Forward	TTTCATCTTCTGTATCAGAGGAACTTATTT	300
	Reverse	ATGGGATGAATCATCAAACAAGAG	300
	MGB probe	ATTATTTTCGTCACGGGTATT	300

*RPP2B*	Forward	TGCTTACTTATTGTTAGTTCAAGGTGGTA	300
	Reverse	CAACACCAACGGATTCCAATAAA	300
	MGB probe	CACCTCTCCATCAGCTT	200

*TDH1 *(*GAPDH*)	Forward	CGGTCCATCCCACAAGGA	300
	Reverse	AGTGGAAGATGGGATAATGTTACCA	300
	MGB probe	AGGTGGTAGAACTGC	200

*PMA1*	Forward	TTGCTTATGATAATGCTCCATACGA	300
	Reverse	TACCCCACAATCTTGGCAAGT	300
	MGB probe	CCAAAACCAGTTAAATGG	200

*LSC2*	Forward	CGTCAACATCTTTGGTGGTATTGT	900
	Reverse	TTGGTGGCAGCAATTAAACCT	900
	MGB probe	AGATGTGATTACGTCGCCA	400

*CPA1*	Forward	TCTGGTGTTGCTGCCATAACTG	300
	Reverse	AATTCTCCCCAATGATGAACCTT	300
	MGB probe	AGACAATTGGTGAGTTATT	200

*IMH*3	Forward	TATTCATATGGCATTATTGGGTGGTA	600
	Reverse	AACCATTTCTGCTTGTTCTTCAGA	600
	MGB probe	TGGTATCATTCATCATAACTGTAC	400

*RIP*	Forward	TGTCACGGTTCCCATTATGATATTT	300
	Reverse	TGGAATTTCCAAGTTCAATGGA	300
	MGB probe	TGGTAGAATTAGAAAGGGTCCA	200

*ALS1*	Forward	CAACAGGCACCTCAGCATCTAC	300
	Reverse	CTCCACCAGTAACAGATCCACTAGTAA	300
	MGB probe	AGTGCTAATAGCGAACTT	300

*ALS3*	Forward	CAACTTGGGTTATTGAAACAAAAACA	300
	Reverse	AGAAACAGAAACCCAAGAACAACCT	300
	MGB probe	AAACTAGCTGTGAAGGTGAT	300
